# Fatigue and Sleep in Airline Cabin Crew: A Scoping Review

**DOI:** 10.3390/ijerph20032652

**Published:** 2023-02-01

**Authors:** Candice C. Y. Wen, Darsh Cherian, Maya T. Schenker, Amy S. Jordan

**Affiliations:** 1 John Trinder Sleep Laboratory, Melbourne School of Psychological Sciences, University of Melbourne, Parkville, VIC 3010, Australia; 2 Institute for Breathing and Sleep, Austin Health, Heidelberg, VIC 3084, Australia

**Keywords:** flight attendant, airline, shift work, fatigue, sleepiness, mental health, safety

## Abstract

Airline cabin crew operate in dynamic work environments that are continuously changing, from unpredictable shift work hours to travelling through multiple time zones. These likely impact cabin crews’ overall health and may affect their performance on safety-related tasks. Research on this population has been limited; therefore, the aim was to summarise the relevant literature regarding fatigue, sleepiness and mental health of cabin crew. This review followed the PRISMA-ScR guidelines and conducted a systematic search utilising five databases. The initial search identified 1223 studies, and through vigorous screening processes, 27 studies were selected for this review. Over half of the selected studies focused on international or long-haul flights, and a large proportion of the sample participants were women. Findings suggested a high prevalence of fatigue and sleepiness as well as unsatisfactory sleep quality with elevated susceptibility to sleep disorders. Factors identified with health outcomes were associated with flight operations (e.g., rosters) and individual differences (e.g., age and coping strategies). Regarding mental health, cabin crews are potentially at a greater risk for depression and anxiety compared to the general public. This review draws attention to the importance of using a standardised approach, such as validated measures for fair and consistent inferences.

## 1. Introduction

In the aviation industry, shift work is considered the norm for the majority of employees. Shift work is associated with numerous adverse mental and physical health outcomes (outlined below). In addition to shift work, the two types of employees who work on aircraft, pilots (also known as flight deck or flight crew) and cabin crew (also commonly known as flight attendants), regularly experience jet lag. This further increases their risk of adverse health outcomes, particularly fatigue and sleepiness. Past research assessing fatigue and sleepiness in the airline industry has focused heavily on the pilot population. Therefore this scoping review aimed to gain a deeper understanding of the literature pertaining to the lesser-studied population: airline cabin crew.

### 1.1. Health Consequences of Shift Work

A large proportion of the working population around the world engages in shift work. Across Europe, 17% of employees were shift workers in 2005, and by 2015 this number increased to 21% [1]. In the United States, 26.6% of employees conducted shift work in 2015 [2], whereas in Australia, in 2021, 16% of the working population were shift workers [3]. Shift workers can be rostered with a permanent or a rotational shift pattern. Permanent shifts, as the name suggests, involve the employee always operating on the same shift schedule. This has traditionally been considered more manageable, as the employee can self-establish a consistent routine to accommodate this. For rotational shifts, the schedule is open to changes. The rotation can be clockwise (e.g., a morning shift followed by an afternoon shift), counter-clockwise (e.g., a morning shift followed by a night shift), or irregular shifts (no pattern to the roster). Rotational shifts are harder to manage with their continuous changes, thus not allowing one to form a routine to support it [4].

Despite the economic benefits that come with non-stop operations around the clock, shift work is not without consequences. Many health problems have been associated with shift work, including elevated risks for cardiovascular diseases [5], diabetes [6,7], obesity [8], sleep disorders [9,10], cancers [11], gastrointestinal issues [12] and poorer mental health [13]. Shift work can be difficult as, more often than not, work is performed out of synchrony with the circadian rhythm. The body’s circadian rhythm operates on an approximately 24 h basis and helps to regulate one’s sleep/wake cycle, cognitive performance and other physiological functions. When performing evening or overnight shift work, it is likely that part of the shift will overlap with one’s habitual sleep time, therefore making it difficult for the employee to stay alert and perform optimally. Further, workers often finish work at a time that coincides with their typical awake time (e.g., daytime) and, therefore, may have difficulty initiating sleep despite feeling tired. It is unsurprising that accidents are far more common amongst shift workers, especially night shift workers-research has found them associated with three times the reported occupational accidents compared to daytime/non-shift workers [14].

Fatigue and sleepiness are common complaints in shift workers. The feelings of fatigue and sleepiness can be similar, and the terms are often used interchangeably under the umbrella of feeling ‘tired’. However, the two are separate, distinct constructs. Fatigue stems from exertion, where one feels lethargic or exhausted from exerting energy or doing work [15]. For example, one may feel fatigued after a run; however, this does not necessitate sleep. Sleepiness, on the other hand, is related to one’s circadian rhythm and homeostatic sleep drive [16]; therefore, the longer one has been awake, the more ‘build-up’ of sleepiness one experiences. Unlike fatigue, the feeling of sleepiness can, fortunately, be relieved with sleep.

Fatigue and sleepiness have been linked to deterioration in both health and performance. The accumulative effect of fatigue and/or poor sleep can lead some shift workers to experience depressed moods, anxiety and stress [13,17,18]. Studies have also found fatigue and sleepiness can reduce cognitive and physical abilities that mimic alcohol intoxication with performance decrements. Moderate levels of fatigue and/or sleep deprivation decrease performance, equivalent to a blood alcohol concentration of 0.05–0.1%, which exceeds the legal alcohol limit in some countries [19,20]. In addition, another study found that loss of 2 h of sleep can negatively impact performance and alertness and increase the risk of errors and accidents [21]. Thus, shift work by itself likely contributes to at least some of the sleepiness, fatigue and poor mental and physical health observed in the aviation industry.

### 1.2. Specific Considerations for the Aviation Industry

Due to the unpredictable nature of flying (e.g., weather changes, air traffic), most pilots and cabin crew have a rotational (i.e., non-permanent) and often irregular roster, which, as noted above, is more difficult to manage compared to a permanent schedule. Pilots and cabin crew both experience the typical consequences of shift work; however, this is further complicated by time zone changes, also known as jet lag. As pilots are in charge of aircraft operations, past research has paid a lot of attention to this occupation, with pilot fatigue being documented as early as 1927 [22]. Past research found pilots regularly experienced fatigue and sleepiness [23,24,25,26], leading aviation policymakers and airlines to install provisions to negate fatigue and sleepiness. For example, controlled rest was implemented on the flight deck to allow a pilot to have a nap or rest (one at a time) during the cruise phase (after take-off, once the aircraft has levelled off and before landing)—this was designed for pilots to temporarily ‘switch off’ and to minimise fatigue [27]. Alternatively, on long-haul flights, two sets of pilots are scheduled to operate the aircraft; one operating and one augmenting crew. This allows one set of flight crew to rest for half of the flight duration and, therefore, reduces the risk of sleepiness during critical phases (take-off or landing).

Despite the extensive research that has been dedicated to pilots, their findings cannot be readily applied to cabin crew. Cabin crew work alongside pilots but have completely different job functions. Firstly, the cabin crew are in charge of the passengers. They are trained to evacuate passengers in an emergency situation, administer first-aid, fight fires, perform safety and security checks, restrain unruly passengers and, of course, provide in-flight services. Secondly, cabin crew have a more physical job and are on their feet for a large portion of the flight, whereas pilots have a greater mental workload and spend the majority of the flight seated inside the cockpit. Thirdly, the majority of the pilots are older and consist of a higher percentage of males, compared to cabin crew, who are more commonly female and have a wider age range with younger mean age than pilots. Cabin crew make up a large proportion of the airline industry; on a commercial flight, there are typically 2–4 pilots operating, whereas the number of operating cabin crew can range from 4–24, depending on the size of the aircraft. Therefore, understanding cabin crew experiences of fatigue and sleepiness is paramount.

In 2005, the United States Congress recognised there are unique issues surrounding cabin crew fatigue and directed the Civil Aerospace Medical Institute to address this. The National Aeronautics and Space Administration (NASA) Ames Research Centre’s Fatigue Countermeasure Group was recruited to investigate these issues further. Their results found most American cabin crews have operated while feeling fatigued and believe this is a common practice in the industry despite the crew appreciating that it is unsafe [28,29]. In America, the passenger workload for cabin crew is roughly one cabin crew member to 50 passengers [30]; thus, each cabin crew bears great responsibility for passenger safety and in case of an emergency. Yet 71% of cabin crew believe their fatigue affected their safety-related performance, and 60% felt their roles in looking after passengers were compromised, both in service and safety [28].

Technical reports from both American and European aviation commissions were interested in factors that may be associated with tiredness. NASA research found more than half of the cabin crew in their study have ‘nodded off’ recently whilst working [28] and identified workload, the work pace and schedule being main contributors. Reports for the European Aviation Safety Agency found that cabin crew reported the most contributing factors to fatigue were ‘long days,’ ‘early starts,’ ‘flying during hours when I would normally sleep,’ and ‘short recovery time between duties’ [31].

Past research that studied both pilots and cabin crew has found nearly 82% of participants have operated flights under the duress of fatigue [32]. This is alarming as only 26.8% felt comfortable enough to file a fatigue report, and two-thirds of the crew suggested no fatigue support was implemented within their airline [32]. A similar result was found with one study reviewing previously submitted fatigue reports, which found an average of 68 cases per 1000 person per year for cabin crew would submit a fatigue report, where 93% of those were stood down or were unable to attend work due to fatigue [33]. It seems the fatigue report was implemented as a solution for cabin crew; however, evidently, few felt encouraged to utilise it. Those who did use the report were in an extremely fatigued state.

With regards to the execution of cabin crew’s fatigue training, the aviation regulation states that fatigue training is mandatory for all crew members. However, retention of this information seems poor, with two-thirds of cabin crew not recalling ever having had fatigue training [28,32]. For those who did recall training, they found it to be useful only to a ‘limited extent’ or ‘not at all’ [28]. Therefore this training may be ineffective.

Although some regions in the world are commissioning technical reports to better understand the effect of fatigue and sleepiness on their cabin crew [28,29,31], to date, there is limited published research investigating this population. Hence, a systematic review is premature, given the current knowledge within the field.

The rationale for this scoping review is, therefore, to establish what is currently known about cabin crew fatigue and sleepiness, summarise current findings and identify gaps and limitations in the literature so future studies can abridge it.

### 1.3. Primary Objectives

1. To summarise what is currently known in the literature on fatigue, sleepiness and other sleep-related constructs in cabin crew;
2. To identify common factors that are associated with fatigue, sleepiness and other sleep-related constructs in cabin crew, e.g., operational factors such as rosters and flight routes or individual factors such as age and gender;
3. To pinpoint existing gaps and limitations in the current literature on fatigue, sleepiness and other sleep-related constructs and to provide suggestions for future directions.

### 1.4. Secondary Objective

4. To summarise what is known about cabin crews’ mental health, specifically depression, anxiety and stress.

## 2. Methods

This scoping review followed the framework of PRISMA-ScR guidelines [34,35] (Supplementary Table S1) and utilised the team method to develop the core concepts to extract data for the review [36]. This scoping review was not pre-registered with its protocol.

### 2.1. Eligibility Criteria

To be considered for the present scoping review, the document must be a published research paper that examined the experience of fatigue and/or sleepiness in commercial airline cabin crew. Further details of inclusion and exclusion criteria are listed in Table 1.

### 2.2. Information Sources & Search

The search strategy was developed in consultation with an experienced librarian to encompass all possible keywords and to search within relevant databases. Table 2 presents the databases and search strategies used. Reference lists from the database search were also screened for relevant papers.

### 2.3. Selection of Sources of Evidence

All findings from the search strategy were considered for abstract reviews. Two reviewers (C.W. and M.S.) independently screened the titles and abstracts. If the decision to keep or discard the abstract was not unanimous, this was resolved with a reviewer discussion on the disagreed abstract. With the shortlisted abstracts, a full article assessment was conducted with two independent reviewers (C.W. and D.C.). Similar to the abstract screening, any disagreement on this was first discussed among the two reviewers; however, when a consensus was not met, a third reviewer (A.J.) was invited to make the final decision.

### 2.4. Data Charting & Synthesis of Results

An excel spreadsheet was created to collate and chart the data. This chart followed the principles of Scoping Review [35] and focused on the three principal components: participant, context and concept. The spreadsheet was initially trialled and created by one author (C.W.) and was further tested by three independent reviewers (D.C., M.S. & A.J.) to confirm its efficacy prior to utilising it for the scoping review.

The following information was collected during the data extraction:

- Article information: author, year of publication, title, journal title, type of study and aim/objective of the study;
- Participant: sample size, cabin crew percentage in the sample, sample characteristics (age and sex), hierarchy and tenure;
- Context: location and type of routes (international versus domestic or long versus short haul);
- Concepts: Outcomes of interest to this scoping review; fatigue, sleepiness, other sleep-related constructs and mental health. With each outcome, the type of measure used, any associated factors and the reported prevalence were examined.

After data charting, information was grouped into one of the following outcomes: fatigue, sleepiness, mental health and relevant measures in search for common results and missing gaps in this area. The key characteristics of cabin crew (age, sex) and airline industry (type of route, sample percentage being cabin crew) were further assessed by their frequency and range.

## 3. Results

### Selection of Sources of Evidence

**Figure 1:**
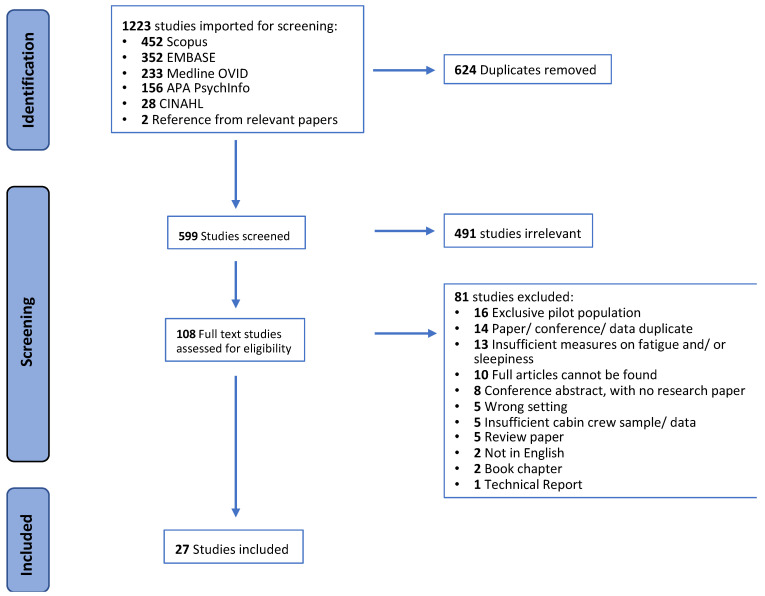
PRISM Flowchart.

Of the initial 1223 studies identified, nearly half of the studies were duplicates. Of the 599 abstracts reviewed, 491 were deemed irrelevant as they did not meet the inclusion criteria (i.e., unrelated participants such as pilots and/or outcome measures such as no sleep or fatigue measure). Subsequently, 108 studies were selected for full-texts assessment, and 27 studies were considered eligible for the present scoping review (see Figure 1). The majority of the studies investigated multiple outcomes of interest; 17 studies looked at fatigue, eight sleepiness, 22 reported other relevant sleep measures and 11 explored mental health issues. One study investigated all four concepts [37]. Table 3 displays the publication details of the studies included in this scoping review.

## 4. Characteristics of Sources of Evidence

### Demographic Characteristics

Within the current scoping review, the study sample size ranged from 19 to 5366 cabin crew (five studies combined cabin and flight crew, and one paper combined cabin crew and teachers). Despite using specific keywords for the database search and stringent abstract screening to attain exclusive cabin crew samples, it is evident that research in this industry is strongly focused on airline pilots. At the full-text review stage, 16 papers were further excluded due to the sample consisting of pilots only, and an additional five papers were not eligible due to insufficient cabin crew sample percentage and/or not reporting results from cabin crew separately (see Figure 1). Of the 27 publications included in this scoping review, 78% of studies (*n* = 21) were entirely conducted on cabin crew, and the remaining studies contained a combination of cabin crew and airline pilots, with two papers meeting the 70% cut-off criteria.

**Figure 2:**
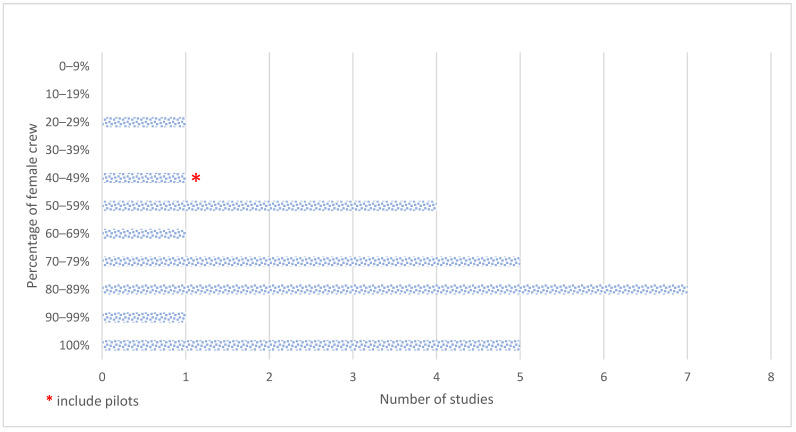
Number of studies with different percentages of female cabin crew in the sample.

Being a cabin crew was traditionally a role predominantly performed by women. However, over the decades, this has evolved to involve both men and women. For this review, in 18 (72%) papers, the sample had more than 70% female participants, suggesting that the literature on this topic is dominated by research on women (see Figure 2).

**Figure 3:**
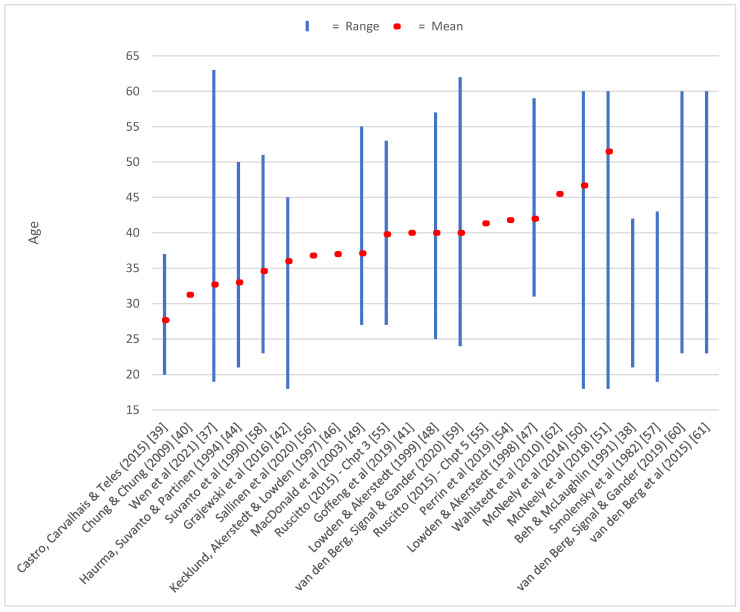
Age mean and range of each paper’s sample. [37,38,39,40,41,42,44,46,47,48,49,50,51,54,55,56,57,58,59,60,61,62].

Despite no restrictions on publication dates, the 27 selected research papers for this scoping review were published between 1982–2021. This suggests the interest in cabin crew fatigue and sleepiness have been of increasing interest in the past decade. Twenty-three studies presented the age breakdowns of their cabin crew sample (mean, range or both). The range of crew within this review was 18–63 years old, with the average mean age of 38.7 years old (see Figure 3).

Twenty-two of the included papers presented the type of routes their cabin crew sample operated in. 54.5% operated in international or long-haul routes, 13.6% in domestic or short-haul and 31.8% serviced both operations or had crew samples from multiple airlines, which encompassed both international/domestic as well as long/short haul routes.

## 5. Concept Characteristics

### 5.1. Fatigue

Measures of fatigue were divided into validated scales, self-designed questionnaires and subscales (see Table 4). Seven papers [37,40,41,55,56,60,61] used four different validated fatigue measures, with the Samn-Perelli Crew Status Check (SP) [63] being the most commonly used tool. Another seven studies used non-validated, subjective fatigue questionnaires [39,43,50,51,53,57,62]. Two fatigue subscales were also used, which were retrieved from Profile of Mood States [64] and Liverpool Jet Lag Questionnaire [65].

### 5.2. Sleepiness

Sleepiness as a construct was not commonly measured in papers identified for this review, with only seven papers (26% of this scoping review) investigating it. Three validated sleepiness scales were used across the seven papers [37,46,47,48,56,60,61]. The Karolinska Sleepiness Scale (KSS) [66] was the most commonly used one and was reported by five of the seven papers (see Table 5). The KSS was developed as a scale measuring sleepiness [66]. However, one study reported this as a measure of fatigue [56]. For the purpose of this review, KSS results will be presented under sleepiness rather than fatigue.

### 5.3. Other Relevant Measures

Thirteen other measures of sleep were considered relevant to this review (see Table 6), including objective measures (i.e., polysomnography, actigraphy, psychomotor vigilance task and neurobehavioural tests), validated scales (sleep quality, insomnia, shift work), subscales measuring sleep, sleep diaries and self-designed subjective questionnaires.

Self-designed subjective questionnaires varied from study to study. However, this method was most commonly used to measure sleep, with 11 studies utilising this method [38,39,41,43,44,46,50,51,55,57,58]. The next most common method was using a sleep/wake diary, with seven papers adopting this [38,41,42,44,46,56,61], followed by actigraphy in six papers [42,47,48,55,60,61].

### 5.4. Mental Health

The secondary objective of this scoping review was to review mental health measures in relation to sleepiness and fatigue, with a particular focus on depression, anxiety and stress experienced by cabin crew. Ten scales and subscales were used between 11 studies [37,40,45,46,48,49,50,51,52,55,57]. Self-designed questionnaires on anxiety and depression were the most commonly utilised method, with three studies adopting this approach [50,51,57], see Table 7.

## 6. Results of Individual Sources of Evidence

### 6.1. Fatigue

Table 4 provides a summary of the main findings from various fatigue measures in the reviewed papers. Fatigue was very commonly reported, with prevalence from validated scales ranging from 63.5% to 77.4% [37,40,55]. The high prevalence of fatigue was considered a health problem [39,43,62] and one of the biggest disadvantages of being a cabin crew member [39]. Compared to the general public, the prevalence of fatigue was double for cabin crew [50], with 21.4–36.8% of cabin crew having sought medical attention due to fatigue during the past 12 months [50,51].

While most measures found a high prevalence of fatigue, the occurrence of fatigue did differ based on the measures used. Interestingly, based on the fatigue subscale (single-item measure) of the Liverpool Jet Lag Questionnaire, nearly two-thirds of the cabin crew did not experiences fatigue [55]. The author raised the validity of a single-item measure; therefore, the results were interpreted with caution. The Chalder Fatigue Scale had two types of scoring processes; Binary and Likert methods. Large discrepancies were reported between the two scoring methods; the Binary scoring found 60.8% of the sample had substantial to maximum fatigue, yet the Likert scoring had only 26.5% reported abnormal to chronic levels of fatigue [55].

This review collated the common factors that were associated with the experience of fatigue. The factors were categorised into flight operations and individual variables.

### 6.2. Flight Operation Variables

Multiple flight operation variables were found to impact the level of fatigue. When reviewing a round trip, inbound flights were associated with greater reports of fatigue than outbound flights [60,61]. The cabin crew reported less fatigue during the pre-flight phase, grew progressively more fatigued during the flight [37,61], and were most fatigued on the commute home [37]. One study asked cabin crew how many hours they were able to work before tiring and found 61.1% would be fatigued from a 4–6 h work day [57]. It was, therefore, not surprising that cabin crew reported being ‘often’ or ‘always’ fatigued at the end of a flight [39,57]. Cabin crew also reported eastward flights ‘wear you out’ more than westward flights, and the subsequent sleep deficits continued into their rest days [49].

The common debate as to which flight route is more tiring (international versus domestic or long-haul versus short-haul) was not clarified by the findings of this review. Some studies found ultra-long-haul flights resulted in greater fatigue as the crew perceived to have greater workloads [60], and long-haul flights were found to be associated with greater health problems due to fatigue compared to short-haul flights [39,43]. Other studies report that being an international cabin crew predicted a higher risk for fatigue [37], and similarly, no fatigue was reported in one study from a short-haul operations cabin crew sample [41]. However, other research concluded that despite international crew having greater scheduled work hours than domestic cabin crew, fatigue was significantly higher amongst the latter [49]. One study explained this with domestic crew often required early morning starts and were scheduled with multiple take-offs and landings per work trip, which elevated fatigue [53]. Another study found fatigue was greater on shorter, non-ultra-long haul inbound flights than on ultra-long-haul outbound flights [61].

There were five studies in this review that compared cabin crew and pilots [41,43,48,52,56]. Fatigue was found to be more commonly reported by cabin crew than pilots [43], and cabin crew believed that airlines provided better support for fatigue management to pilots than cabin crew [59]. Through focus groups, cabin crews reported the overall safety training was excellent; however, the group found the fatigue management training they received to be insufficient [59]. In terms of hierarchy, one study found pursers (i.e., the highest-ranked cabin crew member) reported fatigue symptoms less frequently than cabin crew members [62].

### 6.3. Individual Variables

Fatigue was found to have a profound impact on cabin crews’ quality of life [40]. Families were often considered a supportive framework; however, through fatigue focus groups, families were reported to be an element of stress as they were considered a competing time demand [59]. With limited time resources, traditionally, women were considered more ‘time poor’ due to childcare and housework. However, the studies that compared genders found no differences between their fatigue levels [43,62]. This was further supported by studies with 100% female participants and other studies with greater male participants. Both reinforced that fatigue was a common and detrimental occurrence, which was not unique to women [49,53,59]. When considering age as a factor, younger age was found to be associated with greater fatigue [62].

## 7. Sleepiness

Although sleepiness was not commonly measured in the papers included in this review, the preliminary evidence was concerning (see Table 5), with one study reporting nearly half of the cabin crew to be ‘sleepy’ over the previous month [37,59]. 

### Flight Operation Variables

Unsurprisingly, sleepiness (KSS scores) was worse on workdays than on rest days [46,48]. Cabin crew were most alert at the beginning of their outbound flights [47,60], and as the trip progressed, KSS scores increased (i.e., sleepiness increased) [47,61]. One study found that whist on layovers, cabin crew reported they had severely impaired levels of alertness due to sleepiness [48]. This elevated sleepiness was experienced for the remainder of the trip and would only start to return to the baseline level around the third recovery day [48].

Similar to the fatigue findings, conflicting results were found for the effect of route type on sleepiness. One study found ultra-long-haul flights increased sleepiness [60], whist another reported sleepiness was worse on shorter, non-ultra-long haul inbound flights compared to the longer ultra-long haul outbound flight [61]. This inconsistency may be explained by the factors found associated with sleepiness being the number of consecutive work days [37], early departure times [46] and the duration of awake time [60], whereby the type of route may not be a factor directly associated with sleepiness.

## 8. Other Relevant Measures

Table 6 provides a summary of the main findings from additional measures that were relevant to this review. Interestingly for cabin crew, the number of time zones travelled was not found to impact time spent in bed [58]; however, 78.1% of crews found it to have affected the quality and quantity of their sleep [57], with nearly 60% of the cabin crew reporting poor sleep quality [40]. Sleep duration was heavily impacted by flight duty, with the average sleep duration for workdays being 4.6 h, which was significantly lower than on rest days (7.2 h) and self-perceived sleep needs of 8.1 h sleep per day [54].

This review collated evidence that suggested cabin crew have poor sleep health. Amongst the general population, dissatisfaction with the quality of sleep has a prevalence between 16% and 21% [67], whereas more than half of the cabin crew reported sleep disturbances in the last year [41], and 27.1% have sought medical attention for it. Research also found insomnia had the greatest negative impact on cabin crew’s work ability [45], and 43.6–57.7% screened positive or at risk for it [37,45]. More than two-thirds of the cabin crew were found to be at risk for shift work disorder [37]. They experienced excessive sleepiness when the work schedule coincided with normal sleep time, yet, once off duty, they had insomnia symptoms of being unable to fall or maintain sleep. Another sleep concern, however, on the other end of the spectrum to insomnia, was a report of very short sleep onset latency. In cabin crew, the time it takes to fall asleep has been as little as 6.37 min, which is close to the cut-off time for excessive sleepiness on the multiple sleep latency test (<5 min) [55].

### 8.1. Flight Operation Variables

When cabin crew were operating a trip, the objective measures of vigilance (PVT) elicited better scores on the outbound compared with the inbound sector [60,61]. Unsurprisingly, attentional performance declined as the flight progressed [61], and with every additional flight sector, reaction times increased [41]. Flight delays were another operational variable that affected cabin crew. Flight delays were associated with poor sleep quality; with every delayed flight event, the likelihood of poor sleep quality increased [37].

The most commonly reported periods for sleep disturbances were the night before duty [41], on layovers [48,55] and the first night upon returning home [48,55]. Sleep tended to improve over subsequent rest days [55]. During layovers, cabin crew preferred to switch to local time despite staying on domicile time would reduce the effect of jet lag, whereby on the last day of layover, nearly three-quarters of the crew slept on a local night [61]. Looking at the effects of early rising, one study [46] reported that crew with early starts (duty commenced before 0630) went to bed earlier than non-early starts (duty commenced after 0830). However, the early start crew still had reduced total sleep time by almost two hours compared to the non-early start crew, and a reduction in polysomnography measured stage 2 and rapid eye movement (REM) sleep.

International cabin crew were found to have a lower quality of sleep and a higher risk for insomnia than domestic crew [37,38,44]. For both cabin crew and pilots, more sleep problems were reported after long-haul flights than short-haul flights [43], with crew who operate ultra-long-haul flights obtaining 6.5 h sleep/day (sometimes three brief sleep periods/24 h), compared to 8 h/day (one solid sleep) for grounded crew [38]. Sleep quality was disturbed, with one-third of cabin crew reporting restless sleep prior to transmeridian flights, and upon the return flight, the number of restless sleep reports doubled [44]. This study further found for a 4-day transmeridian flight, it took, on average, 4 days to achieve full recovery in both sleep length and quality [44]. Flight directions were also found to have an impact, with eastward flights affecting cabin crew more negatively than westward flights [38,44]. Eastward travels were linked with greater sleep disturbances, longer sleep latency, lowered sleep quality, difficulty in waking and increased napping [48].

Some studies in this review compared cabin crew with pilots or teachers to gain further insight into this population. Despite both working on aircraft, the comparisons between pilots and cabin crew really highlighted their differences. Specifically, cabin crew had worse PVT performance on average than pilots [61] and reported higher mean values across all health complaints (the most prevalent were sleep problems and tiredness) [52]. Two studies found cabin crew to have, on average, 1 h less sleep than pilots. One of the studies found that 1 h sleep difference started from the night before the first duty day, and the findings suggested the reduced sleep may be due to earlier reporting times required for cabin crew compared to pilots [41]. Similar results were found in another study for rest days, where cabin crew slept on average 6.5 h per 24 h compared to pilots of 7.5 h of the same route [61]. Differences were also found with napping opportunities onboard, where on long night flights, 20% of cabin crew (vs 31% pilots) engaged in on-duty napping. Likewise, on short night flights, 8% of cabin crew (vs 11% pilots) napped. Overall, cabin crew had fewer opportunities to nap than pilots [56]. Comparing cabin crew with a non-shift working profession with a similar demographic (i.e., teachers), cabin crew, on average, slept longer than teachers (7.3 h versus 6.6 h). However, their nocturnal sleep was significantly impaired, with worse sleep efficiency and more wake-after-sleep onset than teachers [42].

### 8.2. Individual Variables

Cabin crew are more likely to have a diagnosed sleep disorder than the general population, with prevalence in the female and male cabin crew being 5.7 and 3.7 times greater, respectively, than in the general population [50]. Inconsistent results were found for gender in relation to sleep quality, whereby one study using actigraphy found women had longer and more efficient sleep than men [48]. Another study using a subjective questionnaire found men reported sleeping more restfully and experienced less sleepiness than women [39]. Age also influenced sleep, with older/higher seniority cabin crew perceiving their sleep quality, adaptation and recovery as worse than younger/less senior cabin crew [58], both at home and away from home [39].

Individual behaviours such as substance use were shown to have an impact on sleep. Studies found the majority of cabin crew (81–91%) consumed caffeinated drinks daily, with a good proportion consuming up to five servings per duty period or within 24 h [37,42,54]. One study found with every 1–2 servings of daily caffeine consumption, sleep quality decreased, and the likelihood of insomnia increased in cabin crew [37]. Further, the use of alcohol was not uncommon amongst cabin crew; compared to teachers, cabin crew were more likely to drink four or more times a week [42]. Another study found 76.4% of cabin crew drank ‘sometimes’ to ‘frequently’ [57], with 41.4% using alcohol to facilitate sleep [37]. Alcohol consumption was further found to be significantly higher on layovers (average 2–3 glasses/day) than at base (average 0.8 glasses) [44]. Drugs (e.g., herbal, cold/flu medication, painkillers, and other over-the-counter medication) have also been used to aid sleep [37,40]. One study found 9.2% of the cabin crew ‘almost always’ or ‘sometimes’ take sleeping pills to facilitate a good night’s sleep [57], with another reporting the use of alcohol and sleep-aid drugs (herbal and over-the-counter) to negatively affect sleep quality and increase the likelihood of insomnia [37].

In the studies that looked at chronotypes, more than half of the cabin crew showed no preference for either morningness or eveningness, and a greater proportion of the remainder tended to have a preference for morningness [39,55].

## 9. Mental Health

Psychological health was one of the domains measured for cabin crews’ quality of life, and it scored the worst compared to the other domains; physical health, social relationships and environment [40]. Fatigue was negatively correlated, and better sleep quality was positively correlated with quality of life [40]. Table 7 summarises the main findings on mental health for this review.

### 9.1. Depression & Anxiety

Mixed results were found for the prevalence of anxiety and depression amongst cabin crew. Two studies found anxiety and depression were relatively uncommon in the samples [45,49]. However, in other studies, a high occurrence or risk of depression was reported. Specifically, female cabin crew were reported to have twice the prevalence of depression compared to the general public, and male cabin crew five times the prevalence [50,51]. In another study, 40% of cabin crew were at risk for depression [37], and 36.3% had a medical diagnosis of depression and/or anxiety [50]. In addition to the high prevalence, the frequency of anxiety symptoms appeared high, with 16.4–20.0% reporting frequent symptoms of anxiety over the past week [50,51], and 14.3% of cabin crew reporting ‘usually’ feeling anxious before a flight, while 36.5% felt this way ‘sometimes’ [57].

Compared to pilots, cabin crew had more reports and higher mean values across the domain of psychological health complaints, including anxiety and depression [52], and international cabin crew had a greater risk for depression than domestic cabin crew [37]. Longer job tenure was found to increase the likelihood of depression and anxiety for both female and male cabin crew [50,51].

### 9.2. Stress

Significantly higher stress was reported for work days compared to rest days [46]. Stress at the workplace was not uncommon, and flying at high altitudes was certainly no exception. More cabin crew reported high levels of work stress compared to pilots; 25% of cabin crew compared to 15% of pilots [52]. Stress was commonly reported for parts of each flight [48], with higher reports of greater stress and mental strain on the inbound flight [48,55], and this elevated feeling of stress would carry across to the first recovery night [48].

## 10. Discussion

**Figure 4:**
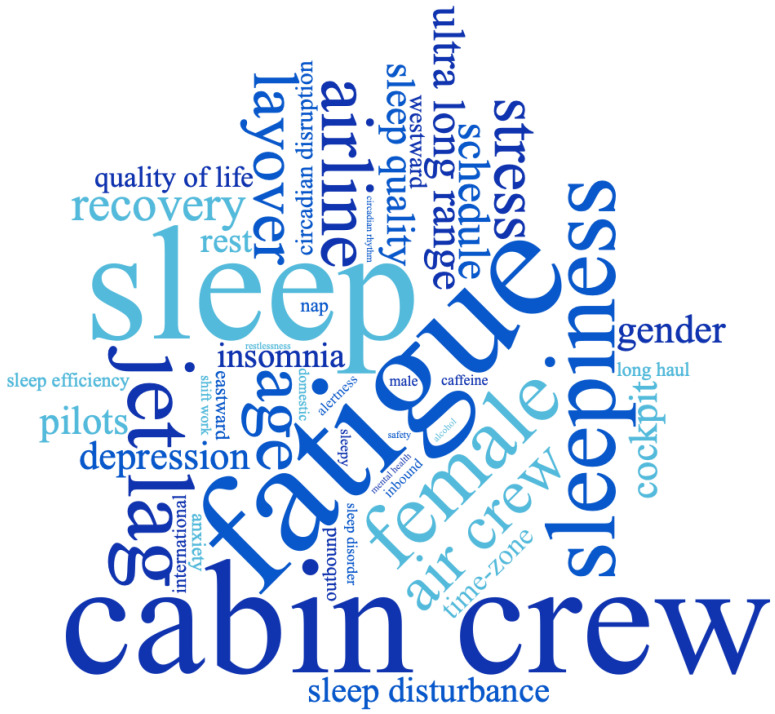
Word Cloud of the current scoping review; summary keywords and common factors.

This scoping review aimed to understand the current literature on cabin crew and their experiences of fatigue, sleepiness and mental health. From analyses of 27 papers, the results found cabin crew had an alarmingly high prevalence of fatigue (ranging from 63.5% to 77.4%), and nearly half of the cabin crew experienced excessive sleepiness. Cabin crew were also found to have poor sleep quality and were vulnerable to sleep disorders, with elevated risks for insomnia and shift work disorder. With regard to mental health, both the symptoms and medical diagnoses for depression and anxiety were higher amongst cabin crew compared to the general public. Common factors relating to fatigue, sleepiness, sleep-related constructs and mental health in airline cabin crew are discussed further below. Figure 4 depicts the current literature in a word cloud [68], with the size of each keyword(s) denoting the frequency retrieved from the 27 abstracts selected for this scoping review.

### 10.1. Flight Operations

Understandably both the experiences of fatigue and sleepiness were low at the beginning of a trip and increased as the flight progressed [37,47,60,61]. For a round trip, inbound flights consistently had greater reports of performance deficits than outbound flights [60,61], and the feeling of fatigue and sleepiness were reported at their peak at the top-of-descent on the inbound flight. Similar results were also found for alertness; the crew were most alert at the start of a trip and steadily declined as the trip progressed [41,61]. Unfortunately, a high proportion of cabin crew experienced fatigue on the commute home (77.4%) [37], and the feeling of sleepiness at the end of a trip was sometimes so severe that some cabin crew had reported falling asleep while driving home [59]. Therefore, not only endangering themselves but others on the road.

For some trips, layovers were implemented for cabin crew to rest before the inbound flight. However, cabin crew did not always capitalise on this opportunity to recharge, and this may partly be explained by jet lag and the constant changes to sleeping arrangements (such as different hotels and cities). This was reflected in the different sleep profiles when at home (average sleep of 7.7 h) compared with away from home (average sleep of 6.5 h) [28]. Despite advice to stay on domicile time, which assists in normalising sleep and reduces symptoms of sleepiness [61], many cabin crews opted not to do this in exchange for the opportunity to sightsee or to dine at local times. This is particularly the case with food, as it may not be readily available if cabin crew stay on domicile time [47]. Cabin crew considered the detriments of staying on domicile time to outweigh the benefits [47].

Understandably, cabin crew are required to work around the clock; however, fatigue complaints were often made on their roster with frequent early start times and late night finishes [39,53]. For domestic cabin crew, early starts and late night finishes were frequently paired together; which not only negatively affected their sleep architecture [46] but it also led to reduced rest periods or rest times at unfavourable circadian phases, which all increased the adverse effect on health [53]. The direction of transmeridian flights was further found to affect fatigue and sleep, with Eastward flights having a greater negative impact on cabin crew than Westward flights. As Eastward flights effectively shorten the day, therefore the ‘local night’ occurs earlier than the body clock is ready for. Thus, with insufficient homeostatic drive, sleep does not come easily, and yet when asleep, the quality is often disturbed [48,58].

With regard to the types of routes cabin crew fly, literature to date has shown conflicting results for both fatigue and sleepiness, suggesting more research could be done in this area. Alternatively, both routes may face the challenges of fatigue and sleepiness; however, their differences may not be easily compared. International or long-haul cabin crew will often travel great distances across multiple time zones per trip. Some may find this type of route to be more tiring as the typical workday often exceeds 12 h, and once at the destination, the crew must juggle with time differences. However, most long-haul flights have scheduled onboard rests and breaks that are built into the flight schedule to alleviate this. Within domestic or short-haul flights, there are different challenges.

Often on a work day, cabin crew operate in multiple sectors, therefore many take-offs and landings. This increases the workload with more passengers to service and multiple safety and security tasks for each flight. For a day with 5–6 flight sectors, cabin crew may look after 700 passengers [41]. Despite no consensus on fatigue and sleepiness with the type of routes, international and long-haul flights were found to affect one’s sleep and increase the likelihood of insomnia. Cabin crew travel multiple time zones annually; one study found the median annual time zone crossed was 93, ranging from 0–465 [42]—although these numbers vary between airlines and regions of the world, this may partly explain the increased risk of insomnia. Another potential explanatory factor may be the continuous changes to sleep environments that would affect one’s sleep on top of the myriad of complications associated with shift work.

Fatigue was found to increase with a greater physical workload [57]. The role of cabin crew changes with their hierarchy, where less physical responsibilities (e.g., delivering in-flight services) are associated with increased seniority. Therefore it was unsurprising that pursers, the highest ranked cabin crew, also known as the cabin crew managers reported the lowest fatigue compared to lower ranked cabin crew. Similarly, this could explain the greater fatigue experienced by cabin crew when compared with pilots; the workload of pilots is predominantly mental compared to the high physical workload of cabin crews. The definition of fatigue is being over-exerted; thus, less energy output from pursers and pilots could explain the lowered fatigue experience.

### 10.2. Individual Differences

No differences were found between gender for their fatigue experience, and results from sleep-related constructs had mixed findings. However, it is worth noting from the papers shortlisted in this review that the majority was dominated by female participants. Thus, the results may be skewed, meaning that conclusions could not be drawn accurately. As for age, the younger cabin crew members had greater reports of fatigue than the older cabin crew. However, as younger crew members are often less junior than the older, more experienced crew, one may question if this relationship was actually associated with age or operational hierarchy. On the other hand, the older/senior cabin crew members were found to have increased sleep disturbance compared to younger/junior cabin crew members. This finding may or may not be unique to cabin crew, as past research has found reduced sleep with ageing [69,70].

Looking at individual behaviours, cabin crews implemented several coping strategies to support their lifestyle. This review’s survey of the literature found high use of caffeine amongst cabin crew [37,42,54] and frequent consumption of alcohol and drugs to facilitate sleep [37,40,57]. Technical reports found that 39% of cabin crew reported using aids to assist them in sleeping when away from home. This ranged from prescription medication, over-the-counter medication and alcohol. Thirteen per cent of the cabin crew reported using at least two methods to achieve sleep [28]. Although not as prevalent, cabin crew also used the listed methods above to gain sleep when at home. Past research has established that caffeine, a stimulant, can increase wakefulness and reduce sleepiness, and alcohol, a sedative, can negatively affect sleep quality. Therefore, these behaviours suggested some cabin crew may be stuck in a vicious cycle due to the use of alcohol to facilitate sleep resulting in bad quality sleep, which subsequently requires more caffeine to stay awake the following day; and have this on repeat. Cabin crews would likely benefit from targeted education on coping strategies that can cast long-term efficiency rather than opting for short-term effects with overall negative consequences.

### 10.3. Mental Health

Mixed results were reported for cabin crews’ susceptibility to anxiety and depression. However, more results indicated cabin crew to be at greater risk for anxiety and depression than the general public, in particular men, with five times the prevalence. Interestingly, this review found international cabin crew had a higher risk for depression [37], and a longer tenure in the airline also increased cabin crews’ risk for depression and anxiety [50,51]. Stress was commonly found to be high amongst cabin crew.

## 11. Limitations

From this scoping review, it was evident that the concepts under investigation lacked consistent measures. The use of validated scales was infrequent, and the most popular method to measure fatigue, sleep-related constructs, and mental health were questions made up by their respective authors. If consistent and validated scales were used, more fair and justified inferences across studies could be made. Another limitation was that, on occasion, fatigue and sleepiness were assumed to be equivalent. For example, the Karolinska Sleepiness Scale (which was designed to measure sleepiness) was used in one study for fatigue [56]. There were several studies that used subscales of broader questionnaires to investigate their aims, understandably, as shorter measures would reduce participant burden; however, unless the subscale has been independently validated, this risks sacrificing the validity of the measure intended.

## 12. Limitations of the Current Review Process

Despite this scoping review following the PRISMA-ScR guidelines [34,35], there are some limitations that may have impacted the overall outcome. Firstly, it is worth noting the potential of author bias. Despite using four different authors across the selection process with stringent criteria, there may be bias from the authors regarding what was considered relevant for this review. For example, the process of selecting which methods to be further assessed under relevant sleep measures could potentially be influenced (for example, which neurobehavioral tests were considered to measure sleep-related constructs). Another limitation is the inclusion and exclusion criteria for cabin crew sample cut-off. This scoping review chose to include the paper for review if the cabin crew sample was more than 70% of the overall sample population. If less than 70%, the paper must present cabin crew data separately from the other occupation. Therefore some pilot results were included in this review. The stringent cut-off criteria or exclusive reports on cabin crew data would have excluded a significant number of papers from being reviewed and reduced the number of studies to be included in the outcomes. Lastly, no restriction on publication dates was included in the search strategy. This could possibly explain the widely varied methods used to measure fatigue, sleepiness, and mental health. With a range of 40 years between the first to last publication included in this review, scales and scoring processes to measure the same constructs have likely changed. Therefore, accurate and direct comparison between studies is difficult.

## 13. Conclusions

Cabin crews across the world are tired, feeling fatigued, frequently experiencing poor sleep, and many are struggling with mental health issues. Due to the nature of the job for cabin crew, good sleep is not consistently achieved, and for some, it is a constant challenge. With flight operations impacting cabin crews’ experiences in fatigue, sleep and mental health, changes could be implemented to better support cabin crew, including more engaging fatigue training or increases in in-flight rests. With many cabin crew adopting coping strategies that are not necessarily beneficial to them (e.g., high caffeine consumption), further education or intervention programs on sleep management would be beneficial. Mental health awareness should also be raised, especially for international and longer-tenured cabin crew-for increased self-awareness and knowledge of how to seek help when needed. Cabin crews play an integral role in ensuring the safety and security of passengers high up in the sky. Therefore, equipping this population with better knowledge and tools to manage their physical and mental health will result in a safer environment for all.

## Figures and Tables

**Table 1:** List of Inclusion and Exclusion criteria for the present scoping review.

	Inclusion Criteria	Exclusion Criteria
Exposure of Interest	Cabin crew of a commercial airline	Other aviation capacities: military, cargo, pilots, ground staff, air traffic controllers
Participants	Research must be cabin crew-focused. All ages and genders included. If other occupations are included in the sample, it needs to fulfil one of two criteria (1) a minimum of 70% of the sample are cabin crew or (2) if less than 70%, cabin crew results must be reported separately to other occupation(s).	Non-cabin crew population. Study sample is not cabin crew dominant (<70% of the sample) and does not report cabin crew results exclusively.
Reported outcomes	Fatigue: Subjective, validated scale, subscale or non-validated measure of fatigue. Sleepiness: Subjective, validated and non-validated measures of sleepiness. Sleep-relevant measures: This includes objective and subjective measures related to sleep, including PSG, actigraphy, PVT, sleep diaries, validated scales, subscales and non-validated measures related to sleep. Mental health: This is a secondary objective. Therefore, one of the three outcomes listed above needs to be fulfilled also. This can be a validated scale, subscale or non-validated questionnaire that examines mental health states.	No outcome measures on either fatigue and/or sleepiness. Outcomes on jet lag and burnout.
Setting	On-the-job measures	Laboratory settings
Language	English	Non-English
Type of Publication	Original Research, Thesis	Review, Conference Abstracts, Book Chapters, Technical Reports
Other	All publication dates and all countries	Full text not available

**Table 2:** Search resources and strategy used for this review.

Search Date	Databases	Search Strategy
20 September 2021	Scopus	“flight attendant” OR “cabin crew” OR “air crew” OR “aircrew” OR “air hostess” OR “airline crew” AND “fatigue” OR “sleep” OR “tired” OR “alert” OR “drowsy” OR “insomnia” OR “lethargic”.
	CINAHL	
	Medline OVID	
	APA PsychInfo	
	EMBASE	

**Table 3:** Research information of the 27 selected studies.

Author (Year)	Title	Type of Study	Main Measures
Beh & McLaughlin (1991) [38]	Mental performance of air crew following layovers on transzonal flights	Observational	Other sleep-related measures
Castro, Carvalhais & Teles (2015) [39]	Irregular working hours and fatigue of cabin crew	Observational	Fatigue Other sleep-related measures
Chung & Chung (2009) [40]	An exploration of quality of life and related factors among female flight attendants	Cross-sectional	Fatigue Sleepiness Mental health
Goffeng et al. (2019) [41]	Risk of fatigue among airline crew during 4 consecutive days of flight duty	Observational	Fatigue Other sleep-related measures
Grajewski et al. (2016) [42]	Sleep disturbance in female flight attendants and teachers	Cohort Study	Other sleep-related measures
Haugli, Skogstad & Hellesoy (1994) [43]	Health, sleep and mood perceptions reported by airline crews flying short and long hauls	Observational	Fatigue Other sleep-related measures
Haurma, Suvanto & Partinen (1994) [44]	The effect of four-day round trip flights over 10 time zones on the sleep-wakefulness patterns of airline flight attendants	Observational	Other sleep-related measures
Hu et al. (2019) [45]	Insomnia, work-related burnout and eating habits affecting the work ability of flight attendants	Cross-sectional	Other sleep-related measures Mental health
Kecklund, Akerstedt & Lowden (1997) [46]	Morning work: Effects of early rising on sleep and alertness	Cohort Study	Sleepiness Other sleep-related measures Mental health
Lowden & Akerstedt (1998) [47]	Retaining home-base sleep hours to prevent jet lag in connection with a westward flight across nine time zones	Quasi-experimental	Sleepiness Other sleep-related measures
Lowden & Akerstedt (1999) [48]	Eastward long-distance flights, sleep and wake patterns in air crews in connection with a two-day layover	Observational	Sleepiness Other sleep-related measures Mental health
MacDonald et al. (2003) [49]	Job stress among female flight attendants	Cross-sectional	Fatigue Mental health
McNeely et al. (2014) [50]	The self-reported health of U.S. flight attendants compared to the general population	Cross-sectional	Fatigue Other sleep-related measures Mental health
McNeely et al. (2018) [51]	Estimating the health consequences of flight attendant work: Comparing flight attendant health to the general population in a cross-sectional study	Cross-sectional	Fatigue Other sleep-related measures Mental health
Omholt, Tveito & Ihlebaek (2017) [52]	Subjective health complaints, work-related stress and self-efficacy in Norwegian aircrew	Cross-sectional	Other sleep-related measures Mental health
Ono et al. (1991) [53]	Working hours and fatigue of Japanese flight attendants	Observational	Fatigue
Perrin et al. (2019) [54]	Timing of Australian flight attendant food and beverage while crewing: A preliminary investigation	Cross-sectional	Other sleep-related measures
Ruscitto (2015) [55]	Predicting jet lag in long-haul cabin crew and making a simple meal plan to ameliorate it—Chapter 5	Thesis chapter: cross-sectional	Fatigue Other sleep-related measures
Ruscitto (2015) [55]	Predicting jet lag in long-haul cabin crew and making a simple meal plan to ameliorate it—Chapter 3	Thesis chapter: Observational and experimental	Fatigue Other sleep-related measures Mental health
Sallinen et al. (2020) [56]	A large-scale European union study of aircrew fatigue during long night and disruptive duties	Cross-sectional	Fatigue Sleepiness Other sleep-related measures
Smolensky et al. (1982) [57]	A health Profile of American Attendants	Cross-sectional	Fatigue Other sleep-related measures Mental health
Suvanto et al. (1990) [58]	Flight attendants’ desynchronosis after rapid time zone changes	Observational	Other sleep-related measures
van den Berg, Signal & Gander (2020) [59]	Fatigue risk management for cabin crew: The importance of company support and sufficient rest for work-life balance—a qualitative study	Focus Group	Fatigue
van den Berg, Signal & Gander (2019) [60]	Perceived workload is associated with cabin crew fatigue on ultra-long-range flights	Observational	Fatigue Sleepiness Other sleep-related measures
van den Berg et al. (2015) [61]	Monitoring and managing cabin crew sleep and fatigue during an ultra-long-range trip	Observational	Fatigue Sleepiness Other sleep-related measures
Wahlstedt et al. (2010) [62]	Psychosocial work environment and medical symptoms among Swedish commercial airline cabin crew	Cross-sectional	Fatigue
Wen et al. (2021) [37]	Health risks and potential predictors of fatigue and sleepiness in airline cabin crew	Cross-sectional	Fatigue Sleepiness Other sleep-related measures Mental health

**Table 4:** Studies of fatigue in cabin crew—the measures used and its main findings.

Validated Fatigue Measures:	Study	Main Findings
Samn-Perelli Crew Status Check (SP)	[37,41,56,60,61]	Cabin crew reported greater fatigue on the inbound flight compared to the outbound [42,43]; Cabin crew reported less fatigue at the pre-flight phase and grew progressively more fatigued during the flight [37,61], and were most fatigued on the commute home (77.4%) [37]; At top-of-descent, fatigue was related to the flight sector, duration of time awake and perceived workload [42]; Factors associated with fatigue during commute home: (1) home travel time; (2) caffeine; (3) Getting picked up after work; (4) Number of sectors/day; (5) Receiving break during trip; (6) flight delay times and (7) ETA between 4–8 am [37]; Mixed results were observed between types of flights; some studies found flying ultra-long-haul flights resulted in greater fatigue when crew perceived their workload as higher [60], and on short-haul operations, fatigue was not reported by either cabin crew or pilots [41]. However, another study found fatigue was worse on the shorter non-ULR inbound flight—indicating longer flight does not necessarily mean higher fatigue [43].
The Multidimensional Assessment of Fatigue Scale	[40]	A total of 76.3% of participants scored moderate-to-high levels for fatigue [40]; For cabin crew, fatigue and sleep quality explained the largest part (16.5%) of the variance in quality of life [40].
The Chalder Fatigue Scale	[55]	On average, a substantial amount of fatigue was reported the day before a flight [55]; however, the scoring process suggested a huge discrepancy: - From binary scoring, 53.2% reported a substantial level of fatigue, with 7.6% reporting the maximum score of fatigue [55]; - From Likert scoring, 72.2% reported ‘normal’ levels of fatigue, 26.5% reported ‘abnormal’ levels, and 1.3% reported ‘chronic’ levels of fatigue [55].
Flinders Fatigue Scale	[37]	A total of 63.5% of the cabin crew were fatigued [37]; Factors associated with fatigue: (1) the number of duty days/week and (2) international crew [37].
Fatigue questionnaires/subscales:		
Focus groups: Fatigue	[59]	The common themes associated with fatigue were (1) insufficient rest; (2) workload; (3) work environment; (4) company support; (5) fatigue management training; (6) self-managing fatigue [59]; Cabin crew reported falling asleep while driving home [59]; Cabin crew reported the fatigue management training was insufficient [59]; Cabin crew believe the airline provides better provisions for fatigue management to pilots than cabin crew despite both groups facing the same challenges [59].
Subjective questionnaire: fatigue-related	[39,43,50,51,53,57,62]	Fatigue was more commonly reported by cabin crew than pilots [43] and twice as prevalent than the general public [50]; Fatigue was considered one of the top disadvantages of being a crew member [39]; more than 50% of cabin crew report fatigue as one of many health problems experienced [43,62], and often or always feel fatigued at the end of flight [39,57]; Female cabin crew have more incidences of health problems than male cabin crew. However, similar levels of fatigue were found between male and female cabin crew [43,62]; Long-haul flights were found with more reports of health problems than short-haul flights; with the greatest differences being related to fatigue [39,43], sleep problems and irritability [43]; A quarter of the cabin crew reported frequent symptoms of fatigue over the past week [50,51]; A total of 21.4–36.8% sought medical attention due to fatigue (over the past 12 months) [50,51]; Fatigue among female cabin crew was 2.18 times and among males 3.04 times as likely to the general public [51]; The most frequently reported factors contributing to fatigue were; long work hours [53], night duty [39,53], the time difference between base and layover destinations [39,53], early starts [53], short recovery time between duties [53] and frequent take-offs and landings for domestic crew [53]; Younger age was found to be associated with fatigue [62]; When asked how many hours they can work before tiring, 10% of crew responded with a maximum of 4 h daily without tiring, and 51.1% responded 5–6 h daily. Thus, 61.1% would be fatigued from a 4–6 h work day [57]; Experiences of fatigue before, during or after flights were most commonly due to the physical requirements of the job (58%), insufficient sleep (26.2%) and emotional stress (7.6%) [57].
Profile of Mood States—Fatigue	[49]	Fatigue was moderately high amongst cabin crew [49]; Despite international crew having greater scheduled work hours than domestic crew (84 h vs 76 h), fatigue was significantly higher amongst domestic cabin crew than international [49]; Cabin crew reported eastward flights ‘wear you out’ more than those in the westward direction, and the consequent sleep deficits continue into nonwork time [49].
Liverpool Jet Lag Questionnaire—Fatigue subscale	[55]	A total of 62.1% were not fatigued, 14.7% were fatigued, and 20% reported some fatigue. The author commented on the validity of using a subscale with single item measure [55]; Fatigue was significantly higher upon landing back home from long haul flight compared to baseline and the subsequent rest days [55].

**Table 5:** Studies on sleepiness in cabin crew—the measures used and its main findings.

Sleepiness Measures	Study	Main Findings
Karolinska Sleepiness Scale (KSS)	[46,47,56,60,61]	Significantly greater sleepiness was reported for flights with early departure times [46,60]; KSS scores were worse on workdays than free days [46], and sleepiness was gradually lowered on recovery days; with self-reported time for recovery being 3 days [47]; Cabin crew were most alert at the beginning of outbound flights [47,60], and as the trip progressed, KSS scores increased to the sleepy part of the scale [47,61]; Crew reported on layovers, whilst staying on domicile time, assisted in normalising sleep and reducing sleepiness; however, this limits leisure activities and food arrangements; these detriments were considered to outweigh the advantages [47]; At top-of-descent, cabin crew reported higher KSS scores more frequently than pilots [56] (note KSS was used as a fatigue measure in this study), and KSS score was associated with the perceived workload as well as the duration of awake time [60]; Cabin crew indicated flying ultra-long-haul flights resulted in more fatigue and sleepiness when they perceived their workload as higher [42]; Sleepiness was worse on shorter non-ULR inbound flights than on longer ULR outbound flights [43].
Accumulated Time with Sleepiness Scale	[48]	Sleepiness ratings increased significantly on flight duty days and increased further on layovers, where approximately 25% of the day reflected severely impaired levels of alertness [48]; Elevated sleepiness was experienced for the remainder of the trip and started to resume baseline level around the third recovery day [48].
Epworth Sleepiness Scale	[37]	A total of 46.9% of the crew reported being ‘sleepy’ over the previous month [37]; Consecutive work days were found to be a factor associated with sleepiness [37].

**Table 6:** Studies with other sleep-related measures used and their main findings.

Objective Measures	Study	Main Findings
Polysomnography	[46]	Prior to working, crew with early starts had reduced total sleep time (almost 2 h) compared to non-early start crew and a reduction in stage 2 and REM sleep [46].
Actigraphy	[42,47,48,55,60,61]	Cabin crew (mean = 7.3 h), on average, slept longer than teachers (mean = 6.6 h); however, the nocturnal sleep is significantly impaired, with worse sleep efficiency and experienced more wake after sleep onset than teachers [42]; At baseline, cabin crew slept on average of 6.5 h/24 h compared to pilots (7.5 h) of the same route [61]; Eastward travels were linked with greater sleep disturbances: longer sleep latency, lowered sleep quality, difficulty in waking and increased napping [48]; Women were found to have longer sleep and more efficient sleep than men [48]; Sleep onset latency for cabin crew was 6.37 min, which is close to the cut-off time for MSLT (<5 min), which correlates with severe sleepiness [55]; Sleep efficiency was negatively impacted on a layover and on the day crew returned home [48,55] and then improved over the next two rest days [55], with increased sleep length, and bedtime advanced significantly [47].
Psychomotor Vigilance Task (PVT)	[60,61]	PVT performed better in the outbound sector than inbound [60,61]; PVT performance declines as the flight progresses (from top-of-climb to top-of-descent) [61]; Compared to pilots, cabin crew PVT performance was worse [61]; PVT performances were worse on shorter non-ULR inbound flights than on longer ULR outbound flights [61].
Attentional Capture Task & Sustained Attention to Response Task	[41]	Cognitive performance did not deteriorate over the 4 work days compared to baseline performance. However, with every additional flight sector, reaction times increased for both cabin crew and pilots [41].
Validated Measures		
Pittsburgh Sleep Quality Index (Global)	[40]	59.9% of participants had poor sleep quality (mean = 6.80 ± 3.23) [40]; For cabin crew, fatigue and sleep quality explained the largest part (16.5%) of the variance in quality of life [40].
Sleep Condition Indicator	[37]	57.7% of the crew were at risk for insomnia [37]; Factors associated with quality of sleep and insomnia were (1) caffeine; (2) alcohol; (3) drugs; (4) home travel time; (5) number of delayed flights; (6) international crew [37].
Athens Insomnia Scale	[45]	Insomnia had the greatest negative impact on cabin crews’ workability, and 43.6% of the crew screened positive [45].
Shift Work Disorder Questionnaire	[37]	A total of 68.0% of cabin crew were at risk for shift work disorder [37].
Survey of Shiftworkers	[54]	The average sleep duration for workdays was 4.6 hrs, which was significantly lower than days off (7.2 h) and self-perceived sleep needs (8.1 h) [54].
Sleep-related measures/subscales		
Liverpool Jet Lag Questionnaire—Sleep subscale	[55]	The cabin crew reported having more difficulties falling asleep and worse sleep quality on the day they landed back home (this includes inbound flight rest) compared to baseline and rest days [55].
Subjective Health Complaints Inventory—Sleep	[52]	Compared to pilots, cabin crew had more reports and higher mean values across all health complaints (the most prevalent were sleep problems and tiredness) [52].
Sleep/wake diary	[38,41,42,44,46,56,61]	International flights were found to increase insomnia either at the beginning or the end of a sleep period; however, the sleep disturbances were more prominent with Eastward than Westward flights [38,44]; Crew operating ultra-long-haul had a mean sleep time of 6.5 h/day (sometimes three brief sleep periods/24 h), compared to 8 h/day (one solid sleep) for grounded crew [38]; The majority of the cabin crew were able to achieve satisfactory sleep before and during their work periods. However, sleep disturbances were commonly reported for the night before the first day of flight duty [41]; Self-reports from sleep diaries found both cabin crew (m = 8.2 h) and teachers (7.4 h) overestimated their undisturbed sleep time by approximately 41 min, compared to actigraphy [42]; The cabin crew with an early start had earlier sleep time on a work day. The wakeup time on a work day is approximately 3 h earlier than the non-early start group and 2.5 h earlier than the free day [46]; On long night flights, 20% of cabin crew (vs 31% of pilots) engaged in on-duty napping, whereas on short night flights, 8% of cabin crew (vs 11% of pilots). Overall, cabin crew had fewer opportunities to nap than pilots [56]; Despite being advised to stay on domicile time, on the last day of layover, 73% of the crew slept during the local night [61]
Subjective questionnaires—Sleep related	[38,39,41,43,44,46,50,51,55,57,58]	For both cabin crew and pilots, long-haul flights had more reports of sleep problems than short-haul flights [43]; A total of 33.7% reported sleep disturbances that were diagnosed conditions by doctors [50]; A total of 27.1% sought medical attention due to sleep disturbance over the past year [51]; Female and male cabin crew had 5.7 and 3.7 times the reported prevalence of diagnosed sleep disorders compared to the general population [50]; Men reported sleeping more restfully and experienced less sleepiness than women [39]; A total of 51% reported sleep disturbances in the last year [41], and 35.4% over the previous week that lasted 5–7 days [51]; The number of time zones travelled did not impact time in bed [58]; however, 78.1% found it to affect the quality and quantity of their sleep [57]; Sleep quality was disturbed by servicing transmeridian flights, with one-third reporting restless sleep prior to flight, and upon the return flight, the number of reports doubled [44]; Older/higher seniority cabin crew perceived their sleep quality, adaptation and recovery as worse than younger/less senior cabin crew [58], both at home and away from home [39]; Cabin crew were found to have, on average, 1 h less sleep than pilots the night before the first duty day; this may be due to earlier reporting time [41]; For a 4-day transmeridian flight, it took, on average, 4 days to achieve the full recovery of sleep length and quality [44]; Despite cabin crew going to bed earlier for the work day, the early-start crew reported insufficient and unrefreshing sleep more frequently and also had greater difficulties waking up to work days than the non-early start crew [46].

**Table 7:** Studies on mental health in cabin crew—the measures used and its main findings.

Anxiety and Depression Measures/Subscales	Study	Main Findings
Brief Symptoms Rating Scale—anxiety and depression	[45]	Normal—79.2%, mild—16.7% and moderate—4.0% [45].
Subjective Health Complaints Inventory—Anxiety & Depression	[52]	Compared to pilots, cabin crew had more reports and higher mean values across psychological health complaints (including anxiety, sadness/depression) [52].
Patient Health Questionnaire -2	[37]	A total of 40.0% of the cabin crew were at risk for depression [37]; International crew were at greater risk for depression than domestic crew [37].
Subjective Questionnaire—Anxiety & Depression	[50,51,57]	A total of 16.3% sought medical attention for depression, and 21.3% for anxiety over the past year [51]; A total of 36.3% have had medical diagnoses of depression and anxiety [50]; Depression amongst female cabin crew is twice the prevalence of the general public, whereas for male cabin crew, it is over five times [50,51]; For female cabin crew, longer job tenure was found to increase the likelihood of depression and anxiety [50], and this was also found with male crew, with each 5-year increase in job tenure [51]; A total of 16.4–20% reported frequent symptoms of anxiety over the previous week [50,51]; Anxiety—14.3% of cabin crew usually felt anxious before a flight, while 36.5% felt this way ‘sometimes’ [57].
Stress and psychological health measures/subscales		
Accumulated Time with Sleepiness Scale—Mood and mental strain	[48]	On the inbound flight, subjects reported greater mental strain [48]; The first recovery night contained elevated feelings of stress [48]; Stress was commonly reported (10–13%) for parts of each flight [48].
Subjective Questionnaire—Stress	[46]	No differences were found between the early and control groups. However, significantly higher stress was reported on work days compared to free days [46].
Profile of Mood States—Psychological distress	[49]	Anxiety, depression and anger were found to be moderately low or low [49].
QPS—Nordic Questionnaire—Stress	[52]	Cabin crew perceived their level of stress as higher than pilots; 25% of cabin crew reported high levels of work stress compared to 15% of pilots [52].
The Stress Arousal Checklist	[55]	Stress was highest on the day of landing back home [55].
The World Health Organisation Quality of Life—psychological health	[40]	Of the four domains measured, psychological health scored the lowest among cabin crew—higher scores represent better QoL [40]; Fatigue was negatively correlated with QoL, and greater sleep quality was positively correlated [40].

## Data Availability

The data presented in this study are available on request from the corresponding author.

## Supplementary Materials

The following supporting information can be downloaded at: https://www.mdpi.com/article/10.3390/ijerph20032652/s1, Table S1: Prisma ScR Checklist.
